# Procalcitonin-guided antibiotic therapy for septic patients in the surgical intensive care unit

**DOI:** 10.1186/s40560-015-0100-9

**Published:** 2015-08-04

**Authors:** John Alfred Carr

**Affiliations:** Department of Trauma and Critical Care, Allegiance Health, 205 N. East Street, Professional Bldg. Ste 203, Jackson, MI 49201 USA

## Abstract

In critically ill patients, elucidating those patients with the systemic inflammatory response syndrome (SIRS) from an infectious source (sepsis), versus those who have SIRS without infection, can be challenging since the clinical features are the same.

Even with strict monitoring and testing, 39–98 % of patients with SIRS will never have bacteriological confirmation of an infection, and 6–17 % of patients with a documented infection will not show signs of SIRS. Due to this overlap, an extensive amount of research has been performed to investigate ways of determining and separating SIRS from infection, compared to SIRS due to trauma, surgical stress, or other non-infectious causes. This review article will discuss the recommended and peer-approved use of procalcitonin in septic patients in the intensive care unit and its use as a guide to antibiotic initiation and termination. The article will focus on the prospective randomized trials (Level 1 evidence) that have been conducted, and lesser levels of evidence will be referenced as needed to substantiate a conclusion.

The literature documents multiple benefits of using procalcitonin as a guide to cost savings and appropriate termination of antibiotics by its use as a new objective marker of bacteremia that was previously not available. This article will show that antibiotics should be terminated when the procalcitonin level falls below 0.5 ng/mL.

## Introduction

In critically ill patients, elucidating those patients with the systemic inflammatory response syndrome (SIRS) from an infectious source (sepsis), versus those who have SIRS without infection, can be challenging since the clinical features are similar [1, 2]. Even with strict monitoring and testing, 39–98 % of patients with SIRS will never have bacteriological confirmation of an infection, and 6–17 % of patients with a documented infection will not show signs of SIRS [3]. Due to this overlap, an extensive amount of research has been performed to investigate ways of determining and separating SIRS from infection, compared to SIRS due to trauma, surgical stress, or other non-infectious causes.

The impetus behind this research is founded, since the mortality rate for patients in septic shock increases by 7 % per hour within the first 6 h of delayed antibiotic administration [4]. And those patients with sepsis from ventilator-associated pneumonia who had a delay of more than 24 h after diagnosis until initiation of antibiotics had a sevenfold higher mortality rate compared to those started on adequate therapy earlier [5]. Thus, it is paramount that patients developing SIRS are quickly screened to ensure an underlying infectious process is not overlooked. The Surviving Sepsis Campaign recommends blood cultures and “imaging studies performed promptly” as screening tools to determine the infectious source [6]. However, blood cultures require days in order to obtain results, and imaging studies are not comprehensive in ruling out an infectious source. This has led to the search for an appropriate serum biomarker to determine and differentiate sepsis from SIRS.

Extensive research looking for the right serum biomarker has covered many potential candidate markers, but the most extensively researched have been interleukin-6, interleukin-8, interleukin-10, C-reactive protein (CRP), and procalcitonin [7–9]. Most of this research has concluded that IL-6, IL-8, IL-10, and CRP are non-specific in separating SIRS due to infection from non-infectious SIRS. However, procalcitonin levels are only mildly elevated in SIRS but become significantly elevated in sepsis and dramatically elevated with Gram-negative sepsis [10, 11]. This finding, although initially reported by Assicot back in 1993, continued to mostly be under-utilized in the care of septic patients until recently, when the research in this area has greatly improved [12].

This review article will discuss the recommended and peer-approved use of procalcitonin in septic patients in the intensive care unit (ICU) and its use as a guide to antibiotic initiation and termination. Due to the voluminous research on procalcitonin and in order to draw appropriate conclusions from the data, this review article will focus only on the use of procalcitonin in septic patients in an ICU setting. Since the procalcitonin level is much higher in septic patients than those with an isolated pulmonary infection, results from these overlapping studies (pulmonary infection without sepsis, pulmonary infection with sepsis, and non-pulmonary sepsis) will be addressed and compared to substantiate the cutoff levels of procalcitonin and its appropriate use as a biomarker of sepsis. The article will focus on the prospective randomized trials (Level 1 evidence) that have been conducted, and lesser levels of evidence will be referenced as needed to substantiate a conclusion.

The literature documents multiple benefits of using procalcitonin as a guide to cost savings and appropriate termination of antibiotics by its use as a new objective marker of bacteremia that was previously not available. This article will show that previous “standard” courses of treatment based upon a pre-set number of 10, 14, or 21 days are no longer appropriate in septic patients and that antibiotics should be terminated when the procalcitonin level falls below 0.5 ng/mL.

## Review

### Molecular physiology

Procalcitonin is the prohormone of calcitonin, but unlike calcitonin which is induced by hypercalcemia, procalcitonin is induced by the activation and adherence of monocytes to the endothelial layer of blood vessels as occurs during sepsis [13, 14]. Unlike cytokines, which can rise and fall due to multiple overlapping and mitigating physiological interactions, procalcitonin release is more tightly controlled and only found in the systemic circulation during response to severe stress and sepsis [14–16]. Multiple research studies have compared procalcitonin to C-reactive protein, cytokines (IL-6, IL-8, IL-10), and lactate, and all of these studies (except one, ref. [17]) have found that procalcitonin was much more sensitive and specific in accurately diagnosing sepsis, and the resolution of sepsis, than any of the other markers [1, 2, 7–9, 12, 18, 19]. In addition, it is useful that procalcitonin levels in normal individuals are very low (<0.1 ng/mL) [14]. Thus, there is substantial evidence that procalcitonin is a reliable marker of sepsis and certainly the best of the biomarkers yet to be identified.

### Prospective randomized trial results

Having established that procalcitonin is a marker of sepsis, the basic science research was supported and upheld by nine prospective randomized trials and refuted by one prospective randomized trial (Tables 1 and 2) [20–29]. Thus, there have been a total of ten prospective randomized trials, and nine have published results (refs. [20–27, 29]), and the tenth trial has closed, but the results have not yet been published at the time of this writing (ref. [28]). It is important to note that not all of the trials were performed in the same patient populations, and therefore, the studies will be grouped and discussed by the population they examined.

**Table 1:** Results of prospective randomized trials

Country	Author	Ref	Trial name	Year	Number	Population	PCT E	PCT C	Number of fewer days of antibiotic use
Use in respiratory infections									
Switzerland	Christ-Crain	[20]	None	2006	302	CAP	0.57	0.44	Yes, 7 days
Switzerland	Briel	[23]	None	2008	458	resp infect	0.8	0.8	Yes, 1 day
Swiss/USA	Stolz	[25]	None	2009	101	VAP	0.6	0.7	Yes, 5 days
Switzerland	Schuetz	[26]	ProHosp	2009	1359	resp infect	0.24	0.24	Yes, 3 days
Use in sepsis									
Switzerland	Nobre	[21]	None	2008	79	Sepsis	8.4	5.9	Yes, 3.5 days
Germany	Schroeder	[22]	None	2008	27	Severe sepsis	7.0	6.0	Yes, 1.7 days
Sepsis in the ICU setting									
Germany	Hochreiter	[24]	None	2009	110	SICU	4.5	4.8	Yes, 2 days
France	Bouadma	[27]	PRORATA	2010	621	ICU	12.0	12.0	Yes, 3 days
Netherlands	De Jong	[28]	SAPS	2013	1816	ICU	–	–	Results pending
Australia	Shehabi	[30]	ProGuard	2014	400	ICU	5.7	8.8	No, 2 days not statistically significant

*Ref* reference number, *n* number of patients enrolled in the study, *CAP* community-acquired pneumonia, *resp infect* respiratory infection, *SICU* surgical intensive care unit, *VAP* ventilator-associated pneumonia, *ICU* intensive care unit, *PCT E* initial procalcitonin level in the experimental group (ng/mL), *PCT C* initial procalcitonin level in the control group (ng/mL)

**Table 2:** Results of prospective randomized trials

Country	Author	Ref	Trial name	Year	Conclusions
Use in respiratory infections					
Switzerland	Christ-Crain	[20]	None	2006	PCT level prevented initial antibx use, earlier discontinuation antibx, less overall use
Switzerland	Briel	[23]	None	2008	PCT level prevented initial antibx use, decreased overall antibx exposure and duration
Swiss/USA	Stolz	[25]	None	2009	PCT level allowed earlier discontinuation antibx, decreased total exposure and duration
Switzerland	Schuetz	[26]	ProHosp	2009	adverse outcomes similar, PCT group had fewer adverse events, less total antibx use
Use in sepsis					
Switzerland	Nobre	[21]	None	2008	PCT allowed earlier discontinuation of antibx, decreased overall antibx exposure
Germany	Schroeder	[22]	None	2008	PCT allowed earlier discontinuation of antibx, cost of antibx reduced
Use in sepsis in the ICU setting					
Germany	Hochreiter	[24]	None	2009	PCT allowed earlier discontinuation of antibx, shorter ICU length of stay
France	Bouadma	[27]	PRORATA	2010	PCT allowed earlier discontinuation of antibx, decreased overall antibx exposure
Netherlands	De Jong	[28]	SAPS	2013	No longer recruiting patients, results pending
Australia	Shehabi	[30]	ProGuard	2014	PCT group had 2 less days of antibx but not statistically significant

*Ref* references, *PCT* procalcitonin, *antibx* antibiotics

### Pneumonia and respiratory infection

Three of the trials enrolled patients with acute respiratory infections or community-acquired pneumonia [20, 23, 26]. One enrolled only patients with ventilator-associated pneumonia and will be discussed separately [25]. These four studies represent pulmonary infection without sepsis. These trials are an important comparative group (without sepsis) to examine against the trials that studied procalcitonin in septic patients.

In all of these trials, an algorithm was followed to determine the initiation and duration of antibiotic therapy based upon serial procalcitonin levels and discontinuation of antibiotics when the procalcitonin level dropped below a pre-determined cutoff level. The three studies examining the use of procalcitonin in guiding therapy for acute respiratory infections and community-acquired pneumonia all reported similar conclusions. All three studies used the same cutoff level of 0.25 μg/L or less as the point to stop antibiotics. And all three concluded that procalcitonin reduced total antibiotic exposure, reduced antibiotic treatment duration, and created similar treatment success in experimental and control groups, and no increased risk was found in stopping the antibiotics once the procalcitonin level normalized. Two of the three also showed that the procalcitonin level was effective in preventing the initial use of antibiotics when the level was initially normal (<0.25 μg/L) [20, 23]. These studies showed a median decrease in antibiotic treatment duration of 7, 1, and 3 days, respectively.

In the one trial studying exclusively those patients with ventilator-associated pneumonia (VAP), the inclusion criteria for diagnosis and the standard treatment of VAP were based upon the previously published guidelines [30]. The procalcitonin cutoff level to stop antibiotics was slightly higher than in the other trials at 0.5 μg/L or less [25]. The authors concluded that procalcitonin allowed a reduction in the overall duration of antibiotic therapy and overall patient exposure to antibiotics. The median decrease in antibiotic treatment was 5 days in the procalcitonin group. The rate of discontinuation of antibiotics overall was also higher in the procalcitonin group. The groups had a similar number of mechanical ventilation days and ICU days. Morbidity and mortality levels were also similar. No increased risk of adverse events was found by discontinuing antibiotics using the procalcitonin-guided algorithm.

### Sepsis in the ICU setting

Four of the prospective randomized trials focused on septic patients (from any source) in an ICU setting, and these trials will be considered together [21, 22, 24, 27]. In all four trials, an algorithm was followed using procalcitonin to guide the duration of antibiotic therapy and to continue antibiotic therapy based upon serially elevated procalcitonin levels until the procalcitonin level dropped below a pre-determined value. At that point, antibiotics were discontinued. Both the procalcitonin group and the control group had a baseline procalcitonin level measured upon initial presentation and inclusion into the study, but only the experimental group had serial procalcitonin levels checked to guide treatment. The control groups were treated by standard courses of antibiotics until the patient was clinically determined to have resolved their septic process.

In all four trials, sepsis was defined appropriately and documented with the Simplified Acute Physiology Score (SAPS II) and the Sequential Organ Failure Assessment (SOFA) score. The rates of microbiologically confirmed sepsis and sepsis severity scores were similar between the procalcitonin-guided and control groups. The median procalcitonin levels on admission to the ICU were similar between the groups and are listed in Table 1.

However, the initial procalcitonin levels are all significantly higher in the sepsis trials and the sepsis-in-the-ICU-setting trials, compared to the respiratory infection/pneumonia trials (Table 1). The procalcitonin levels in the sepsis-ICU-setting trials ranged from 4.5 to 12.0 ng/mL compared to 0.24–0.8 μg/L in the respiratory infection/pneumonia patients. Clearly, the septic patients in the ICU setting were more toxic, and this is reflected in the higher procalcitonin levels. While some authors report procalcitonin levels in microgram per liter (μg/L) and some report the levels in nanogram per milliliter (ng/mL), the reader should be aware that these are, in fact, the same amount, i.e., 1 ng/mL equals 1 μg/L.

The procalcitonin cutoff level for discontinuation of antibiotics was different in all of the ICU-setting trials. In two trials, the cutoff level for discontinuation of antibiotics was <1 ng/mL or less than 35 % of the initial value within 3 days [22, 24]. This level was similar in the two studies because the Schroeder study [22] appears to be a subgroup analysis of the same patients during the same time period by the same authors as the Hochreiter study [24].

In the other two trials, the cutoff level for discontinuation of antibiotics was <0.25 μg/L or a drop of more than 90 % of the peak level in one trial and <0.5 μg/L or a drop of more than 80 % of the peak concentration in the other [21, 27]. What the appropriate cutoff level should be will be discussed later in the manuscript.

Despite the differences in what value would determine discontinuation of antibiotics, all four trials drew similar conclusions. In all studies, the duration of antibiotic treatment was significantly shorter in the procalcitonin-guided groups. The median reduction in antibiotic treatment days was 3.5, 1, 2, and 3 days, respectively [21, 22, 24, 27]. Overall antibiotic exposure was also less in the procalcitonin-guided patients. And none of the trials showed an increase in adverse events or treatment failure rates due to fewer antibiotic days. The Schroeder study also concluded procalcitonin significantly reduced the cost of antibiotics by 18 %, and the Nobre trial showed a statistically significant shorter ICU length of stay [21, 22]. Cure rates, morbidity, and mortality rates were similar between the groups in all of the trials.

One significant concern with a procalcitonin-guided algorithm to stop antibiotics is that a persistently septic patient will have antibiotics discontinued prematurely, resulting in recurrence of infection or other sepsis-related morbidity. This issue was addressed in two of the trials [21, 27], and their secondary endpoints are worth noting in terms of recurrence of infection. The Nobre trial showed that in those patients with a positive blood culture who were assigned to the procalcitonin arm and received a shorter course of antibiotics, no case of recurrent infection was observed after the procalcitonin level had dropped below the cutoff level (0.25 μg/L) [21]. Similarly, in the PRORATA trial, in the procalcitonin-guided arm, no deaths were recorded as related to relapse of infection after discontinuation of antibiotics (cutoff level 0.5 μg/L) [27].

In the PRORATA trial, 28 patients with clinical signs of SIRS assigned to the procalcitonin arm, and 15 patients assigned to the control arm, had initial levels <0.5 ng/mL and were therefore not treated with antibiotics at inclusion into the study. Of the 28 in the procalcitonin arm, eight patients showed a subsequent increase in their procalcitonin level and were later given antibiotics by day 5 (20 patients were never given antibiotics), and there was one death on hospital day 18 from comorbidities. The remainder of the patients did well. Of the 15 patients in the control arm not given antibiotics at inclusion and not having serial procalcitonin levels checked, eight patients were later treated with antibiotics (within 5 days due to clinical deterioration) and four died. The authors concluded that, in terms of mortality, the procalcitonin arm was non-inferior to the control group [27].

Unlike the four prospective randomized trials in a sepsis-ICU-setting mentioned above, there was one prospective randomized trial performed in ICUs in Australia which had negative results [29]. Called the ProGuard trial, this trial was different from the other four ICU-setting trials because the procalcitonin cutoff level to stop antibiotics was set very low at 0.1 ng/mL. This is a normal value [14]. The authors found that with a cutoff level of 0.1 ng/mL, the procalcitonin group did have a median of 2 less days of antibiotics, but this was not significant (*p* = 0.58) [29]. The authors concluded that a procalcitonin algorithm using a 0.1 ng/mL cutoff does not achieve a significant (>25 %) reduction in antibiotic duration.

### Determining the appropriate level to discontinue antibiotics

As the ProGuard trial proved, finding the appropriate cutoff value to stop antibiotics is the yet-to-be-determined critical piece of information that is currently unknown. The Nobre trial showed that a cutoff level of 0.25 μg/L was safe to stop antibiotics, since no recurrent infections were reported after stopping antibiotics at that level [21]. However, a higher cutoff level of 0.5 μg/L was also shown to be safe, with no recurrent infections reported at that level, and that level also allowed a significant reduction in antibiotic usage [27]. With the cutoff level sets too low at 0.1 ng/mL, as in the ProGuard trial, no significant benefit was observed.

Based upon the findings of all of these prospective randomized trials combined, the appropriate cutoff level to stop antibiotics in septic patients in an ICU setting appears to be a serum concentration of procalcitonin of <0.5 ng/mL. This number was successfully used as the cutoff level to stop antibiotics in two of the prospective trials [25, 27], and two trials that used an even higher level of 1.0 ng/mL also reported successful outcomes, although this level is not as frequently cited, and considered too high by other researchers [22, 24]. The 0.5 ng/mL level was also successfully used as an initial threshold not to start antibiotics upon enrollment in three trials [25–27]. With antibiotics withheld at this range, the patients still had successful outcomes.

In order to add more support to using a level <0.5 ng/mL as the cutoff level to stop antibiotics in septic ICU patients, it is helpful to also outline what the literature shows as regards the procalcitonin level that becomes diagnostic, or at least more indicative, of sepsis. The cutoff level to stop antibiotics needs to be well below the diagnostic level at which sepsis is confirmed.

### The procalcitonin level diagnostic of sepsis

In addition to the prospective randomized trials already discussed, there have been an additional 25 prospective non-randomized studies investigating the appropriate level of procalcitonin that is diagnostic of sepsis in ICU patients [9, 18, 31–53]. Since the procalcitonin level has been shown to be consistently higher in septic patients, than those with only bacteremia, respiratory infections, or pneumonia, the ability to find an appropriate diagnostic range is enhanced in this population. And therefore, the results of those studies that focused on patients in the emergency department setting, medical wards, and outpatients have been excluded.

The results of those studies that investigated the procalcitonin level diagnostic of sepsis in ICU populations are shown in Table 3 with the procalcitonin levels separated in groups of ≤0.5, 0.6 to ≤1.0, 1.1 to ≤1.5, and 1.6 to 2.0. The highest sensitivities to detect sepsis were found in those studies that made the diagnosis of sepsis based upon a procalcitonin range of 2.0 ng/mL or higher [45–49, 51–53]. In these studies, the sensitivity to detect sepsis ranged from 65 to 96 %, although six of the studies found the sensitivity to be greater than 86 % [45, 47, 48, 51–53]. In those same studies, the specificity ranged from 70 to 89 % [45–49, 51–53]. As one would expect, as the diagnostic level is dropped down to 1.5 ng/mL or 1.0 ng/mL or less, the overall sensitivity drops as well (Tables 3 and 4) [9, 18, 31–44].

**Table 3:** Procalcitonin diagnostic levels for sepsis in prospective studies in ICU populations

Name	Year	Number	Ref	PCT <0.5	0.6 to <1.0	1.1 to <1.5	1.6 to 2.0	Sensitivity (%)	Specificity (%)
Al-Nawas	1996	337	[32]	0.5				60	79
Bossink	1999	200	[33]	0.5				65	58
Sudhir	2011	100	[34]	0.5				94	–
Ugarte	1999	205	[35]		0.6			68	61
Muller	2000	101	[36]		1.0			89	94
Aouifi	2000	97	[37]		1.0			85	95
Ruokonen	2002	208	[38]		0.8			68	48
G-B	2002	119	[39]		1.0			95	65
Whang	1998	29	[40]			1.1		67	80
Oberhoffer	2000	242	[41]			1.4		84	83
Wanner	2000	405	[42]			1.5		76^a^	77
Harbarth	2001	78	[9]			1.1		97	78
Tugrul	2002	85	[43]			1.3		73	83
Hensler	2003	137	[44]			1.5		42^a^	73
Castelli	2004	150	[18]			1.1		79	85
Mokart	2005	50	[45]			1.1		81	72
Brunkhorst	2000	185	[46]				2.0	96	86
Suprin	2000	101	[47]				2.0	65	70
Meisner	2002	208	[48]				2.0	87	78
DeTalance	2003	108	[49]				2.0	91	89
Luzzani	2003	70	[50]				2.0	76	84
Du	2003	51	[51]				1.6	80	74
Geppert	2003	55	[52]				2.0	87	75
Endo	2008	82	[53]				2.0	95	78
Meynaar	2011	76	[54]				2.0	97	80

^a^These studies included trauma patients, PCT is known to rise after trauma unrelated to sepsis

*ref* reference

**Table 4:** Content and references

Subject	References
Prospective randomized trials	[20–28, 30]
Discontinuing antibiotics	[20–28]
Diagnosing sepsis	[9, 18, 32–54]
Differentiating sepsis and SIRS	[10, 18, 20–24, 26, 27, 30, 55–57]
Monitoring sepsis	[16, 17, 19, 31, 58–61]

With this in mind, another review of Table 1 displaying the five prospective randomized trials in septic patients that were conducted in an ICU setting shows that the initial procalcitonin level documented for the septic patients in those trials ranged from 4.5 to 12 ng/mL. Thus, the diagnostic level of 2.0 ng/mL appears to be appropriate to diagnose sepsis in an ICU population, with an acceptable sensitivity and specificity.

### Differentiating sepsis from SIRS

The third piece of information to be discussed is differentiating sepsis from SIRS. Although there is significant evidence supporting elevated procalcitonin in septic patients, procalcitonin levels are also mildly elevated in SIRS without infection. Several studies have addressed areas of overlap between the two.

It has been documented from the outpatient versus ICU-setting trials that the initial level of procalcitonin will be higher in a patient with sepsis compared to one with only a respiratory infection or bacteremia (Table 1). In addition, procalcitonin is not elevated in patients with a simple localized infection. While the three outpatient prospective randomized trials examining the usefulness of procalcitonin in respiratory infections did show true benefit, the procalcitonin levels were not highly elevated, and the differences between starting levels, peak levels, and the cutoff levels were quite small [20, 23, 26].

This is because every patient with an infection is not septic, and procalcitonin has shown its real value in determining when to stop antibiotics in septic patients in an ICU setting. All of the prospective randomized ICU trials showed significant elevations in the procalcitonin levels in septic patients [21, 22, 24, 27, 29]. Two observational studies that examined the differences in procalcitonin levels in patients with documented sepsis compared to SIRS found that the median level in septic patients ranged from 1.58 to 29.3 ng/mL, while in the SIRS group, the median levels were 0.38–0.74 ng/mL [10, 18]. Another study that examined baseline procalcitonin levels in patients with end-stage renal disease on hemodialysis without infection found that as their baseline inflammatory state reflects, the procalcitonin levels in these patients remained mildly chronically elevated at a median range of 0.50 ± 0.49 ng/mL [54]. A study that investigated the procalcitonin levels in patients with neurotrauma without infection found that, depending upon the severity of the brain injury, the procalcitonin levels were mildly elevated from 0.05 to 0.13 for mild brain injury, 0.11 to 0.55 for moderate injury, and 0.17 to 0.79 for severe brain injury [55]. Overall, these studies have concluded that most patients with non-infectious SIRS maintain an inflammatory-mediated procalcitonin level from 0.3 to 0.8 ng/mL [10, 18, 54–56].

### Using procalcitonin to monitor for sepsis in the ICU: static levels versus trends

Although older research showed that procalcitonin levels in sepsis do not correlate with mortality [17], more recent work has shown that increased mortality was documented for patients with pneumonia whose levels exceed 2.5 ng/mL and in septic trauma patients whose levels exceed 5 ng/mL [19, 57]. In addition, a persistent elevation in the procalcitonin level, or no decrease in the level after antibiotic treatment, has been shown to correlate with a higher mortality rate [58–61].

Thus, it is important to continue to follow the trend of the procalcitonin level while the patient is in the ICU. The level can change, and certainly, a patient initially admitted to the ICU may subsequently develop an infection, or later become septic, and the procalcitonin is a useful guide to follow. A dramatic increase in the procalcitonin level should certainly raise suspicion of an infection or worsening of an ongoing septic process. It has been recommended that if the clinical picture is indicative of sepsis, and the initial procalcitonin level is low, that antibiotics should be started anyway, and repeat procalcitonin levels checked at 12, 24, and 36 h until the diagnosis is clear [16].

Having now established that a level ≥2.0 ng/mL is most sensitive and specific for sepsis and that a level <0.5 ng/mL is safe to stop antibiotics in septic ICU patients, there is a persistent “gray area” from 0.5 to 1.9 ng/mL. Most patients with SIRS have levels of 0.3–0.8 ng/mL, which occupies some of this range. For now, these levels can only represent worsening or resolution of an inflammatory or septic process, depending upon which way the trend moves. Thus, the trends are very important and will need to be followed as the patient worsens or improves.

Of course, procalcitonin is only a biomarker of sepsis, and clinical judgment is still primary in deciding upon initiation and termination of antibiotics. However, there is now substantial evidence that procalcitonin can successfully be used to guide antibiotic therapy and adds tremendous benefit in limiting the excessive use of antibiotics. This biomarker is much more physiologically reliable as an objective marker of ongoing infection, and its use is justified by prospective randomized data. A procalcitonin-based algorithm to terminate antibiotics should be more widely accepted and is superior to courses of antibiotics based upon a pre-determined number of days.

## Conclusions

The following conclusions use the Oxford Centre for Evidence-Based Medicine 2009 levels of evidence:

1. In septic ICU patients, a clinically recovered physiological situation and a serum procalcitonin level less than 0.5 ng/mL appear to be an acceptable and safe time to discontinue antibiotics. (Level 1b evidence)
2. The use of procalcitonin to decide when to stop antibiotics based upon a level less than 0.5 ng/mL in patients with pulmonary infections and/or sepsis has been shown to reduce total antibiotic use and decrease the duration of antibiotics. (Level 1b evidence)
3. An appropriate clinical situation and a procalcitonin level above 2 ng/mL are diagnostic of sepsis with a high sensitivity and specificity, and antibiotic therapy should be started immediately. (Level 2a evidence)
4. A patient with a systemic inflammatory response and a procalcitonin level less than 0.5 ng/mL is very unlikely to have an infectious etiology of the SIRS response, and antibiotics can be withheld, although the procalcitonin level should be trended. (Level 2a evidence)

## Abbreviations

CRP: C-reactive protein
ICU: intensive care unit
L: liter
mL: milliliter
Ng: nanogram
PCT: procalcitonin
SIRS: systemic inflammatory response syndrome
μg: microgram
